# A Metabolites Merging Strategy (MMS): Harmonization to Enable Studies’ Intercomparison

**DOI:** 10.3390/metabo13121167

**Published:** 2023-11-21

**Authors:** Héctor Villalba, Maria Llambrich, Josep Gumà, Jesús Brezmes, Raquel Cumeras

**Affiliations:** 1 Department of Oncology, Hospital Universitari Sant Joan de Reus, Institut d’Investigació Sanitària Pere Virgili (IISPV), CERCA, 43204 Reus, Spain; 2 Department of Electrical Electronic Engineering and Automation, University of Rovira i Virgili (URV), 43007 Tarragona, Spain; 3 Department of Nutrition and Metabolism, Institut d’Investigació Sanitària Pere Virgili (IISPV), CERCA, 43204 Reus, Spain; 4 Department of Medicine and Surgery, University of Rovira i Virgili (URV), 43007 Tarragona, Spain

**Keywords:** metabolites, harmonization, merging, studies intercomparison, InChIKey

## Abstract

Metabolomics encounters challenges in cross-study comparisons due to diverse metabolite nomenclature and reporting practices. To bridge this gap, we introduce the Metabolites Merging Strategy (MMS), offering a systematic framework to harmonize multiple metabolite datasets for enhanced interstudy comparability. MMS has three steps. Step 1: Translation and merging of the different datasets by employing InChIKeys for data integration, encompassing the translation of metabolite names (if needed). Followed by Step 2: Attributes’ retrieval from the InChIkey, including descriptors of name (title name from PubChem and RefMet name from Metabolomics Workbench), and chemical properties (molecular weight and molecular formula), both systematic (InChI, InChIKey, SMILES) and non-systematic identifiers (PubChem, CheBI, HMDB, KEGG, LipidMaps, DrugBank, Bin ID and CAS number), and their ontology. Finally, a meticulous three-step curation process is used to rectify disparities for conjugated base/acid compounds (optional step), missing attributes, and synonym checking (duplicated information). The MMS procedure is exemplified through a case study of urinary asthma metabolites, where MMS facilitated the identification of significant pathways hidden when no dataset merging strategy was followed. This study highlights the need for standardized and unified metabolite datasets to enhance the reproducibility and comparability of metabolomics studies.

## 1. Introduction

Metabolomics comprehensively analyzes small molecules, metabolites, in any biological system [1], which, with the rapid development of high-throughput analytical technologies, has enabled the measurement of thousands of metabolites [2]. However, the diversity of metabolites, their chemical complexity, and, most importantly, how different authors report them make it challenging to annotate and compare metabolite data generated by different laboratories or platforms [3]. A data harmonization process involves integrating and standardizing data from various sources to ensure that they can be effectively compared and analyzed. The goal is to make the data coherent and compatible, allowing researchers, analysts, and decision-makers to work with a unified and consistent dataset. This involves addressing issues such as data format differences, naming conventions, data quality, and data structure variations. Data format, quality, and structure will be specific to each source used. The RefMet reference nomenclature for metabolomics [4] was proposed to overcome the naming convention issues in metabolomics. Also, the inclusion of different kinds of identifiers will aid in the usability and comparability between different datasets’ results and can be divided into systematic and non-systematic chemical identifiers.

Systematic chemical identifiers are names assigned to chemical substances according to a set of rules established by an international body. These names are designed to be unique and unambiguous, and they provide a standardized way to represent chemical structures. Examples of systematic chemical identifiers include the International Union of Pure and Applied Chemistry (IUPAC) names, which are based on a set of rules for naming organic and inorganic compounds, and the International Chemical Identifier or InChI [5], which encodes the chemical structure of a compound into a unique sequence of characters. InChI is designed to provide a comprehensive and unique identifier for a chemical compound, which can be used for database management, chemical search, and comparison. While InChI primarily focuses on the structural aspects of a molecule, such as the arrangement of atoms and bonds, it has some limitations [5]. These limitations include: (i) limited representation of stereochemistry, as a more accurate depiction of cis/trans isomerism and other stereochemical features is needed; (ii) handling complex chemical scenarios, such as mixtures, polymers, complex chiral chemicals, and inorganic compounds; (iii) limited ability to represent multiple structures, such as antibodies with hundreds of amino acids, handling tautomeric forms, representation of metal bonds, and complex mixtures with varying stoichiometries, positional isomers, and variable bonding situations; (iv) limitations in representing proton moieties [6]. Despite these limitations, InChI has become a canonical identifier for the communication of well-defined chemicals and has greatly simplified the linking and aggregation of most content in public structure-centric chemical databases. Dashti et al. introduced ALATIS [7], an adaptation of InChI unique compound identifiers that rely on three-dimensional (3D) structures, allowing precise atom labeling in small molecules. The ALATIS naming convention has been adopted by the BMRB small molecule NMR archive [8] and the NMReData initiative [9], making it particularly valuable for NMR-based harmonization studies. It has also been employed to verify InChI consistency in databases like PubChem [10], unveiling non-standard InChI strings, inconsistent atom labeling, and inaccurate cross-references. However, the specialized focus of ALATIS on 3D structures and individual molecule identification may limit its suitability for the more extensive and intricate task of harmonizing diverse metabolite datasets, which encompasses broader objectives beyond database use.

The other types of identifiers used are non-systematic chemical identifiers which, on the other hand, are not based on a standardized set of rules and may be ambiguous or inconsistent across different databases. These identifiers are often assigned by individual researchers or organizations and may include common names, trivial names, or proprietary codes or identifiers. Examples of non-systematic chemical identifiers include PubChem Compound IDs, HMDB IDs, and KEGG IDs, which are unique identifiers assigned to each compound in their respective databases. Akhondi et al. [11] investigated the ambiguity of non-systematic chemical identifiers within and between eight widely used chemical databases. The central objective of the study was to quantify the extent of this ambiguity associated with the identifiers and to systematically assess their consistency within and between studies. The study revealed that while ambiguity within individual datasets is generally low, non-systematic identifiers shared among databases exhibit higher levels of ambiguity, leading to potential inconsistencies in associated compound structures. This underscores the complexity of validating non-systematic identifiers, often necessitating manual verification.

Given the challenges faced in metabolomics regarding the variation in metabolite nomenclature and reporting practices, addressing the issue of non-systematic chemical identifiers requires the implementation of a comprehensive strategy. Several databases have been developed to store and curate metabolite information, such as the Human Metabolome Database (HMDB) [12], BinVestigate [13], Marker DB [14], COCONUT [15], KNApSAcK [16], and BinDiscover [17], among others. However, each database has strengths and limitations, such as differences in data content, format, and accessibility. Merging multiple metabolite datasets implies integrating data from distinct sources, such as web-specific databases (e.g., HMDB, BinVestigate, Marker DB, COCONUT, KNApSAcK, BinDiscover, etc.), literature (application and user specific), and repositories (e.g., Metabolomics Workbench [18], MetaboLights [19], GNPS [20], among others). In addition, harmonizing metabolite identifiers is essential to facilitate the integration of metabolite data from different sources. In this regard, the IUPAC InChI and standard InChI hashes (InChIKey) [5] are systematic chemical identifiers widely used in metabolomics research due to their unique representation of molecular structures. The InChIKey is a hashed version of the InChI that is shorter and more convenient to use in databases and search engines. The InChIKey is used by several databases to identify and link chemical structures to other resources. For example, the UniChem database [21] provides a large-scale non-redundant database of pointers between chemical structures and chemistry resources using InChIKey. The US-EPA CompTox Chemicals Dashboard [22] also uses InChIKey to support non-targeted analysis and high-throughput toxicity testing. The HMDB [12] uses InChIKey to encode the chemical structure of metabolites.

Despite all the existing tools, the practice of merging information from metabolomics studies remains scarce within the scientific community. Researchers have predominantly relied on two primary strategies to address this challenge. The first strategy involves the integration of data from a single platform over time, with the objective of ensuring consistent compound identification [23]. The second strategy, when opting for a cross-platform approach, relies on the straightforward merging of compounds based solely on their names [24,25]. These two strategies represent the prevailing approaches to merging metabolomics data, but they are not without their limitations. While they have been instrumental in some cases, they may not always be sufficient to fully harness the potential insights that can be derived from metabolomics data integration. Hence, there is a need to explore additional methods and techniques that can enhance the merging of metabolomics information, ultimately advancing our understanding of complex metabolic processes.

Here, we present the Metabolites Merging Strategy (MMS) that enables the cross-platform and cross-technique integration of multiple metabolite datasets. The aim of the MMS is to obtain reliable datasets of metabolites with several identifiers to enable databases’ and studies’ intercomparisons. The first step of MMS is to translate all metabolite names to systematic identifier InChIKeys and merge them. Second, attributes for each InChIKey are obtained, which include: (a) descriptors: chemical name as PubChem, RefMet accepted name and chemical properties (molecular weight, molecular formula, InChI, SMILES); (b) non-systematic identifiers: PubChem ID (CID), HMDB ID, Bin ID, KEGG ID, LipidMaps ID, DrugBank ID, CAS number, ChEBI ID); and (c) ontology. The third and last step is to perform a manual curation to check for conjugated base/acid compounds, missing attributes, and duplicated information. To usability of the MMS is exemplified by the case study of urine asthma metabolites, which included 10 sources of information (three studies from Metabolomics Workbench, the Human Metabolome DB, and six journal articles), obtaining a merged dataset with 391 metabolites. Finally, an enrichment analysis shows the importance of using the MMS, as two pathways are found significant (FDR < 0.05), in contrast to when the MMS is not followed and no significant pathways are returned.

## 2. Materials and Methods

For data wrangling, a data editor was used. For attribute translation, application programming interface (API) connectivity or representational state transfer (REST) services were used with Python (version 3.10.6) to extract the data whenever possible (Table 1). First, InChIKey was obtained from compound names via the PubChem Identifier Exchange Service [26] or from identifiers other than InChIKey via the Chemical Translator Service (CTS) [27], and results were merged. Second, attributes were retrieved from: PubChem Identifier Exchange Service [26] (Name (as “title”), PubChem ID or CID, InChI, InChIKey, SMILES); Metabolomics Workbench [28] (RefMet accepted name); Chemical Translator Service (CTS) [27] (molecular weight (MW), molecular formula, CheBI ID, HMDB ID, KEGG ID, LipidMaps ID, DrugBank ID, CAS number); BinVestigate ID or Bin ID [13]; and with InChI as input, ClassyFire [29] (ontology). Third, a final three-step manual curation was carried out to: (1) convert conjugated base/acid compounds to the non-conjugated compounds; (2) retrieve missing information; and (3) check for duplicates.

For the used case study, ten input data were used from three different sources (see Table 2): (i) one repository, the Metabolomics Workbench [18], with three studies: ST001039 (study I), ST001048 (study II), and ST001317 (study III); (ii) one database, the Human Metabolome Database (HMDB) [12]; and (iii) six literature articles (LT01–06). Metabolomics Workbench was filtered by species “human”, sample source “urine”, and disease “asthma”. The HMDB was filtered by biospecimen locations “urine” and associated disorders and diseases “asthma”. The literature was searched in PubMed with the keywords: “urine asthma metabolomics”.

Enrichment analysis was performed with MetaboAnalyst 5.0 [30], with pathways based on the Small Molecule Pathway Database (SMPDB) [31], for the compounds found in more than two sources.

**Table 2:** Case study sources used, including repository studies, databases, and literature articles.

Source	Reference	Named As	Instrument (Column for LC-MS)
Repository	Metabolomics Workbench ST001039	Study I	LC-MS (HILIC, RP C18)
Repository	Metabolomics Workbench ST001048	Study II	LC-MS (HILIC, RP C18)
Repository	Metabolomics Workbench ST001317	Study III	LC-MS (HILIC, RP C18)
Database	Human Metabolome Database	HMDB	GC-MS, LC-MS, NMR
Literature	Carraro S et al., 2018 [32]	LT01	LC-MS (RP HSST3)
Literature	Chiu CY et al., 2018 [33]	LT02	NMR
Literature	Chiu CY et al., 2020 [34]	LT03	NMR
Literature	Li S et al., 2020 [35]	LT04	GC-MS
Literature	Li J et al., 2022 [36]	LT05	LC-MS (RP HSST3)
Literature	Tao JL et al., 2019 [37]	LT06	GC-MS

## 3. Metabolites Merging Strategy

**Figure 1:**
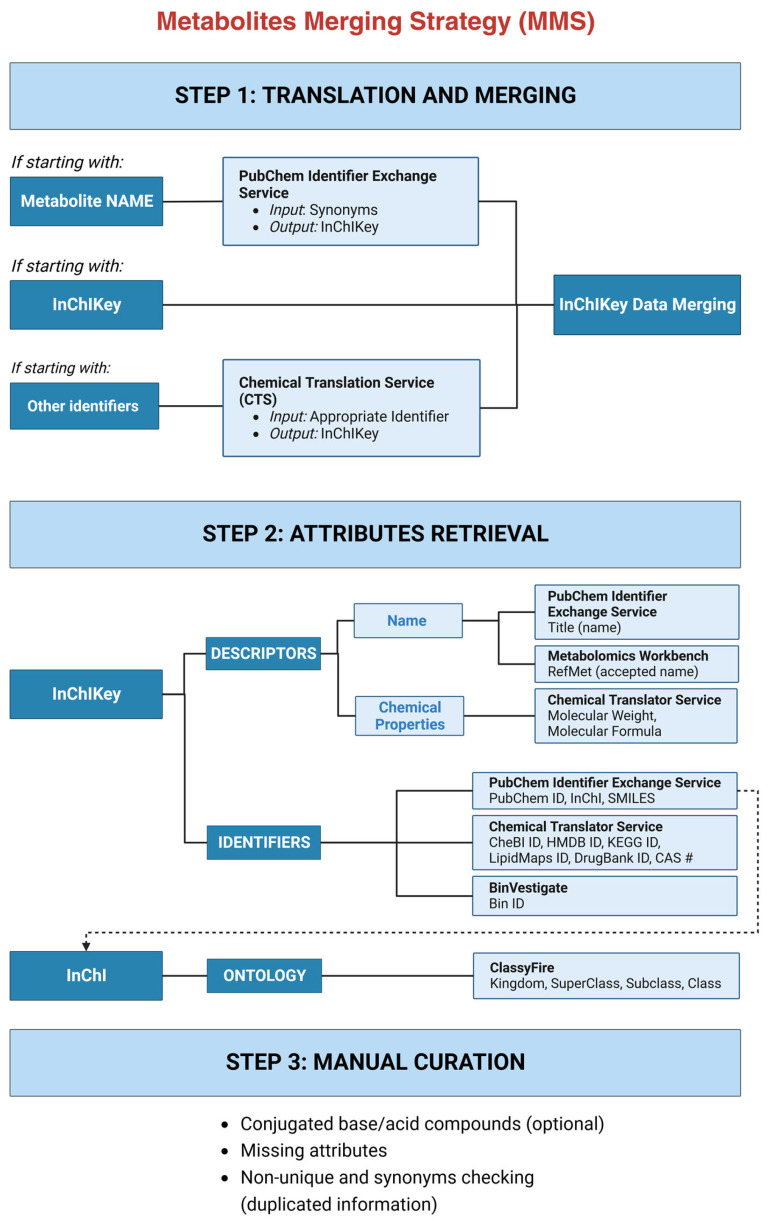
MMS three-step roadmap. STEP 1: Translation and Merging, enabling the conversion of diverse dataset inputs to InChIKey identifiers. STEP 2: Attributes’ Retrieval to enrich compound representations through compounds descriptors, identifiers, and ontology. STEP 3: Manual Curation, including (optional) conjugated base/acid compound conversion, missing attributes’ retrieval, and comprehensive attribute-based and name-based curation. Created with BioRender.com.

The proposed Metabolites Merging Strategy has three main steps (Figure 1), that will enable the conversion of all different datasets’ inputs to a unique identifier (InChIKey) via Step 1 (translation and merging), the retrieval of several identifiers via Step 2 (attributes’ retrieval), and finally a finished dataset after a three-step manual curation process via Step 3 (manual curation).

### 3.1. MMS Step 1: Translation and Merging

The first step of MMS is translating all metabolites from the individual datasets to the International Chemical Identifier (InChIKey) and subsequently merging them. The InChIKey enables efficient integration and comparison of the data. Three situations can happen as initial conditions for the harmonization process in different datasets: names are reported, and/or InChIKeys are reported, and/or other identifiers are reported. If names are reported, the PubChem Identifier Exchange Service is used to retrieve the InChIKey. If other identifiers than InChIKey’s are reported, then the Chemical Translation Service is used to retrieve the InChIKey. Once InChIKeys have been retrieved, data are merged. Duplicated InChIKey entries are combined ensuring that the source is included in the “Reference” column of the merged dataset.

### 3.2. MMS Step 2: Attributes’ Retrieval

The second step of MMS is to retrieve metabolites’ attributes. Attributes are characterizations that increase the number of representations for one single compound. There are descriptive attributes in which the information flows in only one direction. For example, starting with an InChIKey we can obtain the molecular weight of a metabolite, but we cannot extract the InChIKey starting from the molecular weight of a metabolite. The attributes that have these characteristics are the ones that provide non-specific chemical properties (molecular weight, molecular formula) and ontology information (subclass, superclass, class, and kingdom). However, in certain attributes (RefMet, HMDB ID, Bin ID, DrugBank ID, KEGG ID, LipidMaps ID, ChEBI, CAS number, PubChem CID) the interchange of information is bidirectional. Therefore, we can obtain a unique InChIKey associated with one of these attributes, and conversely, we can obtain the same attribute starting from the InChIKey.

Attributes can be divided into three different groups (Figure 1): (1) descriptors’ attributes including metabolite name (from PubChem and RefMet) and chemical properties (molecular formula, MW); (2) identifier attributes that will allow the user to easily find the metabolites of interest in several databases (InChI, InChIKey, SMILES, CID, CheBI, HMDB, KEGG, LipidMaps, DrugBank, CAS, and Bin); (3) ontology attributes.

### 3.3. MMS Step 3: Manual Curation

The last step is a three-step manual curation procedure. The first (optional) manual curation step is to convert the conjugated base/acid compounds to their non-conjugated form, by using the information contained in the InChIKey name. The first 14 characters of the InChIKey encode the molecular skeleton, whereas the second part encodes the stereochemistry and the isotopes. We recommend carrying out this step for conjugated base/acid compounds rather than using it for all isotopes and stereoisomers, as then isotopes, specific 3D arrangement of the atoms, different stereoisomers (such as cis–trans isomers), or other stereochemical details will be lost, which for researchers working with chiral LC-MS or NMR will not be recommendable. Conjugated base/acid compounds will be deleted ensuring that the source is included in the “Reference” column of the non-conjugated compound. Second, missing attributes are retrieved (if possible), by checking them on PubChem and the Chemical Translator Service, as it sometimes fails when batch conversion is used. This step is strongly recommended, as the compound identifiers and descriptors can aid the user during the curation process. Finally, non-unique and conflicting synonyms (duplicate entries) are checked for all bidirectional attributes (including the name and RefMet name and all the identifiers). In case of a duplicate, entries are combined ensuring that the source is included in the “Reference” column of the merged dataset.

## 4. Case Study: Urinary Asthma Metabolites

For the intercomparison of studies of urinary asthma metabolites, three studies were retrieved from Metabolomics Workbench, specific metabolites were retrieved from one database (HMDB), and six reported studies were retrieved from the literature. Following the MMS, a total of 547 metabolites have been retrieved and merged, leading to a final dataset of 391 metabolites (Supplementary Table S2). The repository studies contributed 135 metabolites from study I (Supplementary Table S3), 234 metabolites from study II (Supplementary Table S4), and 183 metabolites from study III (Supplementary Table S5). The HMDB had 13 additional metabolites associated with asthma in urine (Supplementary Table S6). And finally, 71 metabolites were retrieved from the six selected articles (Supplementary Table S7).

**Figure 2:**
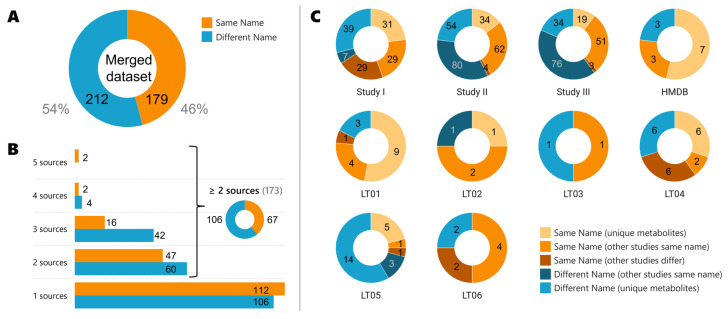
Case study results. (**A**) Merged dataset of the 10 sources studied combined. Color code: metabolites reported with the same name (orange), metabolites reported with different names (blue); (**B**) Number of compounds found in the different sources: repositories (studies I–III), databases (HMDB), and literature (LT01–06); (**C**) Individual dataset amounts. Color code: metabolites with the same name unique to the study (light orange), metabolites with the same name that are found also in other studies (orange), metabolites with the same name that are labeled differently in other studies (dark orange), metabolites with different names but that are named correctly in other studies (dark blue), and metabolites with different names (blue).

In the merged dataset, 179 metabolites (46%) are reported with the same name, while 212 (54%) are reported with different names (see Figure 2A). As explained in the Materials and Methods section, the name was selected from PubChem name title, therefore, when we say “same name” it means the compound is reported in the repository, databases, or literature reports with the PubChem name title. Similarly, when a “different name” is stated, it means the reported name is not the PubChem “title” name. Even though we checked 10 sources of information (3 Metabolomics Workbench studies, the HMDB, and 6 literature articles (LT01–06)), only two metabolites have been found in 5 sources of information (see Figure 2B): stearic acid and uric acid. Stearic acid (IUPAC systematic name: octadecanoic acid, abbreviated as C18:0) is a saturated long-chain fatty acid (C_18_H_36_O_2_) present in many animal and vegetable fats and oils. In asthmatic patients, stearic acid has been found to be significantly decreased in asthmatic bronchial smooth muscle [38]. On the other hand, uric acid (IUPAC name: 7,9-dihydro-3H-purine-2,6,8-trione, abbreviated as UA) is a xanthine. Uric acid is a product of the metabolic breakdown of purine nucleotides; in fact, it is the last product of purine metabolism in humans, and it is a normal component of urine. Serum levels of uric acid (SUA) have been associated with asthmatic patients during an acute exacerbation [39]. SUA is one of the non-enzymatic antioxidants during an asthma exacerbation [40]. It acts as a protective mechanism against lung damage by large amounts of oxidants. 

Of the 173 metabolites that are found in at least two different sources of information (see Figure 2B), only 67 (39%) have the same name reported. We have analyzed the individual sources of information (Figure 2C) and detailed if the compound was reported with the same name or if it differed, but also if the compound was unique to the study, and if the compound was found in the other studies analyzed with the same or a different name. This has led to five subgroups labeled as “same name (unique metabolites)”, “same name (also in other studies)”, “same name (other studies differ)”, “different names (other studies correct)”, or as “different name”. By following the MMS, 65 compounds of study I were merged instead of the 29 if the MMS was not followed (65/29). These 65 account for 58 of the “same name” compounds not unique to the study, plus 7 “different name (other studies correct)”. Similarly, 146/62 in study II, 130/51 in study III, and 28/14 if all articles are combined (LT01-06) were merged with/without following the MMS. In total, if we sum the “same name (other studies differ)” and “different name (other studies correct)” groups, this would lead to 213 compounds out of 372 that would not have been merged if the MMS was not followed.

Even though the number of compounds with the same name title as PubChem is higher in almost all sources analyzed, the number of compounds with different names between studies varies largely from one specific study to another (Figure 2C). For example, study I has 89 metabolites that have the same name title as PubChem, while 46 metabolites had different names. Interestingly, other studies had a higher number of metabolites with different names, e.g., 134 for study II of which 80 are found correctly in other studies, or 110 for study III of which 76 are correct in other studies. The HMDB reported 77% of the metabolites to have the same names (10 of 13). For the literature studies analyzed, only the reported significant metabolites have been added for each study, and 60% of the metabolites (45 of 75) have the same name if all literature articles were combined (LT01–LT06). 

## 5. Discussion

The Metabolites Merging Strategy (MMS) described here implies a notable advancement in the field of metabolomics. Its significance lies in bridging the knowledge gap of those unfamiliar with studies’ harmonization, thereby promoting standardization in the field. Built on the premise that the integration of metabolomics data from diverse sources can yield a more comprehensive view of the metabolome, MMS transcends the constraints of individual studies, enabling a deeper and more precise comprehension of the metabolites present in biological samples. The MMS involves three steps. First, it integrates metabolomics data from diverse sources, by standardizing the representation of metabolites with the systematic and standardized InChIKey identifier. Second, to allow the results’ intercomparisons and a better dataset curation, several attributes are retrieved. In the same line, a metabolomics standard reporting strategy has been proposed by Alseekh et al. [41], which includes multiple information from each compound to ensure cross-study merging. The third and final step involves the dataset manual curation, to rectify disparities for conjugated base/acid compounds (optional step), missing attributes, and for synonym checking (duplicated information). Once the MMS has been followed and the final dataset generated, a final data interpretation step will be followed, where the merged information will provide a more comprehensive understanding of the metabolome, enabling researchers to draw meaningful biological insights from the integrated data. 

The MMS has been successfully implemented for urinary asthma metabolites. The merged dataset includes complete information regarding chemical properties (molecular weight and molecular formula), the systematic identifiers InChIKey, InChI, and SMILES, the non-systematic identifier PubChem ID (CID), and the ontology (for kingdom and superclass). Other non-systematic identifier attributes are partially covered: CAS number (99%), RefMet accepted name (95%), CheBI ID (88%), HMDB ID (87%), KEGG ID (83%), BinVestigate ID (42%), DrugBank ID (42%), and LipidMaps ID (21%). The variation in percentage coverage of these non-systematic identifiers can be attributed to the specific scope and content of each database. The observed differences emphasize the challenge of harmonizing data from diverse sources with distinct foci and methodologies. For example, BinVestigate is a database of GC-MS data only, reducing the number of metabolites it contains with respect to those detected by LC-MS. On the other hand, DrugBank provides information about compounds used in drug formulation, and LipidMaps is a lipid-specific database. 

The distribution of metabolite names within the merged dataset bears notable implications for the comprehensive integration and harmonization achieved by the proposed MMS. The observation that 46% of the metabolites are reported with the same name across different sources highlights the presence of a substantial level of consistency in nomenclature. However, improvement is needed. If researchers start to adopt the use of systematic chemical identifiers, such as InChIKeys, along with the name of the metabolites, it will provide a standardized and unambiguous representation of molecular structures and will ease the intercomparison of studies. Such uniformity in nomenclature not only facilitates efficient data integration but also minimizes the potential for errors stemming from discrepancies in metabolite naming. However, the significant proportion of metabolites (54%) reported with different names points to the persisting challenge of diverse metabolite nomenclature practices. This heterogeneity in naming conventions is indicative of the complexities inherent in metabolite annotation, arising from factors such as historical context, variations in reporting practices, and differences in data curation methodologies across different sources. As a result, the MMS’s capability to merge data with differing metabolite names acquires heightened significance, as it bridges gaps in data integration that could otherwise hinder meaningful cross-study comparisons. 

Of the 173 metabolites that are found in more than one study, 106 metabolites (61%) differ in their naming between studies. Three situations are found for metabolites with different names. First, the use of conjugated forms like palmitate instead of palmitic acid. To target these cases, we checked the first 14 characters of the InChIKey (27 characters long). They inform of the connectivity and tautomeric representation of an InChI string and, consequently, offer insight into the metabolite structure. The second part contains 10 characters that are related to all other InChI layers (isotopes, stereochemistry, etc.). The duplicated entries are combined keeping the non-conjugated ones. Second, the use of a synonym like 3,4-dihydroxycinnamic acid from LT04 instead of caffeic acid. Finally, the use of acronyms. For example, CAR 12:0 from ST001039 is referred to as lauroylcarnitine in ST001048, whose PubChem title name is in fact O-dodecanoylcarnitine. A critical aspect of the MMS is the manual curation step, which aims to ensure data accuracy and completeness. This step involves checking for conjugated compounds, addressing missing attributes, and resolving duplicated information. Manual curation remains essential due to the intricacies of metabolite data, highlighting the need for human expertise in validating and refining integrated datasets.

**Figure 3:**
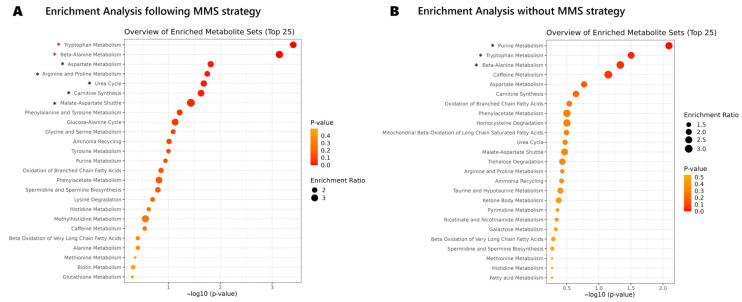
Enrichment analysis results: (**A**) following the MMS; and (**B**) without following the MMS. Asterisk * indicates *p* < 0.05. Red asterisk indicates FDR < 0.05.

Without the implementation of the MMS, the merged dataset, instead of 391 entries, would have 547 entries and data analysis would lead to different results and misinterpretations. We conducted an enrichment analysis for the metabolites found at least in two sources, wherein we evaluated their overrepresentation in specific biological functions or pathways to unveil potential insights into their functional significance. If the MMS was followed (Figure 3A), seven pathways exhibited relevance (*p* < 0.05) and two pathways demonstrated significance (false discovery rate (FDR) < 0.05), specifically, tryptophan and beta-alanine metabolism pathways (Supplementary Table S7). Conversely, when the MMS merging procedure was not employed (Figure 3B), three pathways were relevant (*p* < 0.05) but none achieved significance (Supplementary Table S8). Tryptophan metabolic pathways have been implicated in asthma’s pathogenesis [42], with metabolites demonstrating the potential to mitigate lung inflammation and airway hyperreactivity [43]. On the other hand, beta-alanine metabolism exhibited higher levels in severely asthmatic patients [44]. The MMS procedure indicated the involvement of these metabolic pathways in asthma, which otherwise would not be found relevant.

The proposed strategy effectively mitigates challenges stemming from inherent biases and variability within repositories, databases, and literature sources through a meticulously designed framework encompassing systematic data harmonization, rigorous verification, and meticulous curation processes. By leveraging InChIKeys as systematic chemical identifiers, the strategy ensures a standardized representation of metabolite structures, facilitating integration and comparability across heterogeneous sources. The translation of metabolite names and subsequent attribute consolidation, encompassing identifiers, chemical properties, and ontology, ensures comprehensive data representation. A rigorous three-step curation process further rectifies disparities, encompassing the resolution of conjugated compounds, retrieval of missing information, and identification of duplicates.

The MMS approach to data harmonization, verification, and curation enhances the accuracy and reliability of the integrated dataset, effectively addressing biases and variability intrinsic to diverse data origins and establishing a robust foundation for cross-study comparisons in metabolomics research. Moreover, the MMS’s adaptability and versatility are evident in its capacity to effectively accommodate a spectrum of data sources encountered in diverse metabolomics studies. The strategy’s applicability extends beyond the presented case study, as it can readily accommodate metabolomics data from repositories, databases, and literature articles characterized by dissimilarities in content, format, and quality. By customizing the attribute retrieval step to align with specific data source characteristics, the MMS can seamlessly handle varied metabolite identifiers and attributes, allowing the user to further use the merged dataset with several other databases or applications. This adaptability positions the MMS as a versatile tool for harmonizing and integrating metabolite data from multifaceted sources, elucidating pathways, and enabling meaningful cross-study comparisons across a spectrum of metabolomics investigations.

## 6. Limitations

The Metabolites Merging Strategy (MMS) offers a valuable approach for integrating metabolomics data; however, it has some limitations. Resulting merged datasets may lack comprehensive coverage of the entire metabolome. Certain metabolites might be poorly represented or incorrectly aggregated due to the dependence on the available data for merging and the specific scientific question(s) being addressed. The breadth of metabolome coverage is intricately tied to the data accessible for merging and the focus of the research. Broad research topics may introduce misconceptions during the data integration process. A second limitation relates to the uses of the systematic InChIKey identifier. InChIKeys may encounter difficulties when dealing with data generated by diverse analytical techniques. In the context of metabolomics, different analytical methods are used to collect data on chemical compounds. Consequently, when merging data from these different analytical techniques, InChIKeys may face challenges in accurately linking and combining chemical compound information, potentially leading to issues in data integration. To overcome this issue, for example, ALATIS [10], designed for predicting 3D atomic structures from NMR data, can be used to provide detailed 3D structural information, particularly when dealing with complex mixtures and stereoisomers. This strategy has been used by the Biological Magnetic Resonance Data Bank (BMRB, https://bmrb.io) [8]. However, the 3D structure of the compounds is not available when using cross-technique public studies’ information, as is the case of the MMS procedure. Moreover, metabolomic databases often lack consistent contextual information about how a metabolite participates in biochemical reactions. Metabolites can have varying roles in biochemical pathways, which are often context dependent. The absence of this contextual information can limit the interpretability of the metabolomics data. Databases primarily focus on storing and providing data about the metabolites themselves, such as their names, structures, and concentrations. They may not always include comprehensive information about the biochemical reactions in which these metabolites are involved. Efforts like MNXref from MetaNetX [45] trying to establish cross-references between metabolites and biochemical reactions facilitate the reconciliation of different biochemical databases and genome-scale metabolic networks. However, integrating multiple omics fields remains a challenge beyond the scope of this article. Finally, the used attributes might not be relevant to every user, given that each study may have its unique specificities and preferences. For instance, certain identifiers like METLIN ID, KnapSack ID, MoNA ID, COCONUT ID, GNPS ID, or CompTox ID from EPA, among others, have not been included in MMS. Researchers will need to choose and employ the attributes that best suit their specific research needs and objectives, and MMS is a first step in achieving a standardized and harmonized approach to metabolomics data integration.

In metabolomics research, the challenge of merging studies extends beyond nomenclature. While establishing consistent nomenclature is undoubtedly a crucial aspect, it is important to recognize that other factors also play a significant role in the successful integration of metabolomics data. Depending on the specific objectives of merging studies, researchers need to give due consideration to various elements, including the study design, participant characteristics, and ensuring the comparability of samples. When employing the Metabolites Merging Strategy (MMS), it is imperative to acknowledge that it is just one piece of the puzzle. To effectively combine metabolomics studies, researchers must address a range of prerequisites and criteria that extend beyond nomenclature alone. Therefore, in this context, we propose a strategy aimed at enhancing nomenclature consistency. However, it is important to emphasize that this should be seen as just one step in a broader process. To achieve a robust and meaningful integration of metabolomics data, researchers must also address the broader aspects of study design, participant selection, and ensuring the compatibility of samples.

## 7. Conclusions

The Metabolites Merging Strategy (MMS) provides a robust and systematic methodology for integrating and harmonizing multiple metabolite databases. The MMS, consisting of three key steps—translation and merging, attributes’ retrieval, and manual curation—provides a structured framework to harmonize metabolite data across multiple studies. The use of a representation of the molecular structures through InChIKeys provides an insightful approach for merging data in different ways, thereby gaining novel insights into biological systems. The presented case study on urinary asthma metabolites demonstrates the utility of MMS in creating a comprehensive merged dataset, despite variations in metabolite names and attributes among different sources. The implementation of standardized naming conventions within the metabolomics community will be key for advancing the field and achieving efficient data integration and harmonization across multiple databases, studies, and reports. Additionally, standardized naming enables the development of comprehensive metabolite databases, facilitating cross-referencing and providing valuable insights into intricate metabolic pathways. Initiatives like RefMet need to be fostered by the community to achieve this goal. The MMS procedure not only contributes to the ongoing efforts to standardize and harmonize metabolomics data integration, facilitating advances in the metabolomics field, but also serves as the foundation for a call to harmonize nomenclature between databases, repositories, and other relevant entities. We encourage the broader community to join us in this endeavor.

## Figures and Tables

**Table 1:** Tools used in the Metabolite Database Merging strategy, including links to the tools themselves or to their API/REST services.

Tool	Link	Input	Output
PubChem Identifier Exchange Service	https://pubchem.ncbi.nlm.nih.gov/idexchange/ (accessed on 26 July 2023)	Name as reported	InChIKey
		InChIKey	InChI, SMILES, CID, name (“title”)
Chemical Translator Service	https://cts.fiehnlab.ucdavis.edu/services (accessed on 27 July 2023)	InChIKey	MW, molecular formula, ChEBI, HMDB, KEGG, LipidMaps, DrugBank, CAS number
Metabolomic Workbench	https://www.metabolomicsworkbench.org/tools/mw_rest.php (accessed on 31 July 2023)	InChIKey	RefMet Accepted name
BinVestigate	https://binvestigate.fiehnlab.ucdavis.edu/rest/bin/ (accessed on 31 July 2023)	InChIKey	Bin ID
ClassyFire	https://bitbucket.org/wishartlab/classyfire_api/src/master/ (accessed on 31 July 2023)	InChI	Ontology

## Data Availability

Datasets are available at the Zenodo repository (10.5281/zenodo.8226097).

## Supplementary Materials

The following supporting information can be downloaded at: https://www.mdpi.com/article/10.3390/metabo13121167/s1, Table S1: Merged urine asthma metabolites; Table S2: Metabolomic Workbench ST001039 (study I) metabolites; Table S3: Metabolomic Workbench ST001048 (study II) metabolites; Table S4: Metabolomic Workbench ST001317 (study III) metabolites; Table S5: HMDB urine asthma metabolites; Table S6: Literature urine asthma significant metabolites; Table S7: Enrichment analysis pathways results following the MMS; Table S8: Enrichment analysis pathways results without following the MMS.
